# Comprehensive MRI-guided intracardiac electrophysiological interventions in swine using a combination of active tracking and passive real-time catheter imaging

**DOI:** 10.1186/1532-429X-16-S1-O49

**Published:** 2014-01-16

**Authors:** Matthias Grothoff, Christopher Piorkowski, Thomas Gaspar, Sebastian Hilbert, Philipp Sommer, Steffen Weiss, Sascha Krüger, Tom Lloyd, Bernhard Schnackenburg, Gerhard Hindircks, Matthias Gutberlet

**Affiliations:** 1 Diagnostic and Interventional Radiology, University Leipzig - Heart Center, Leipzig, Germany; 2 Department of Electrophysiology, University Leipzig - Heart Center, Leipzig, Germany; 3 Department of Electrophysiology, University Dresden - Heart Center, Dresden, Germany; 4 Innovative Technologies, Philips Technology GmbH, Hamburg, Germany; 5 Research & Development, IMRICOR, Burnsville, Minnesota, USA

## Background

Electrophysiological (EP) procedures guided by MRI have the potential of improving catheter navigation and of characterizing both the arrythmogenic substrate as well as ablation induced tissue changes. Here we present the results of a series of navigation, mapping and ablation studies using a combination of active catheter tracking and real-time catheter imaging.

## Methods

A 3D data set containing heart and thoracic vessels was acquired in 8 swine (37-42 kg) using a 1.5T MR-scanner and a breath hold 3D-whole-heart-sequence. An advanced MR-EP-platform (iSuite, Philips Research Hamburg) created auto-registered 3D-models of all cardiac chambers. Two MRI conditional steerable diagnostic and ablation catheters (Vision, Imricor Medical Systems) were inserted via femoral sheaths. Active catheter tracking was performed using the magnetic field to localize inductive coils assembled on the EP-catheter. The coils were shown as a virtual catheter icon displayed in real-time in the auto-segmented/auto-registered 3D-model, in the pre-acquired MRI planes, and during further scanning. The positions of the catheter tip were confirmed by fully balanced steady-state-free-precession (SSFP) sequence with a frame rate of 8 per second. Initially the CS was intubated. After transseptal access the catheter was brought into all PVs. Subsequently bi-atrial SR activation map was acquired. Ablation procedures were performed alternately in the PVs, the cavotricuspidal isthmus and the posterior wall of the right atrium. For visualization of the myocardial edema and necrosis a T2-weighted turbo-spin-echo and a 2D-T1-weighted phase sensitive inversion recovery sequence was used respectively. After RVOT access the AV-node was ablated, the pig sacrificed and the explanted hearts inspected for ablation lesions.

## Results

The protocol could be completed in all swine with a mean procedural time of 114 ± 18 min. using active tracking mostly. The intubation of the CS was peformed within a mean time of 11.6 ± 11.3 min. by active tracking only in one pig, in all others by additional passive real-time imaging. Transseptal puncture was successful with a mean procedural time of 16.4 ± 10.1 minutes. In one pig a non-MR-compatible puncture set had to be used. Bi-Atrial mapping was time-efficient. The position of the catheter tip as visualized by active tracking could reliably be confirmed with passive real-time imaging. No complications occurred. For the detection of the different lesions the fused images with the ablation side labeling tool of the iSuite Software was very helpful.

## Conclusions

The combination of active catheter tracking and passive real-time visualization in MR-guided EP-studies using advanced interventional software like the iSuite was safe and enabled efficient navigation, mapping and ablation. A set of standard interventional EP-procedures could be successfully performed. However, there are still limitations before this technique can be considered as an alternative to standard fluoroscopy-guided EP-procedures.

## Funding

No funding.

**Figure 1:**
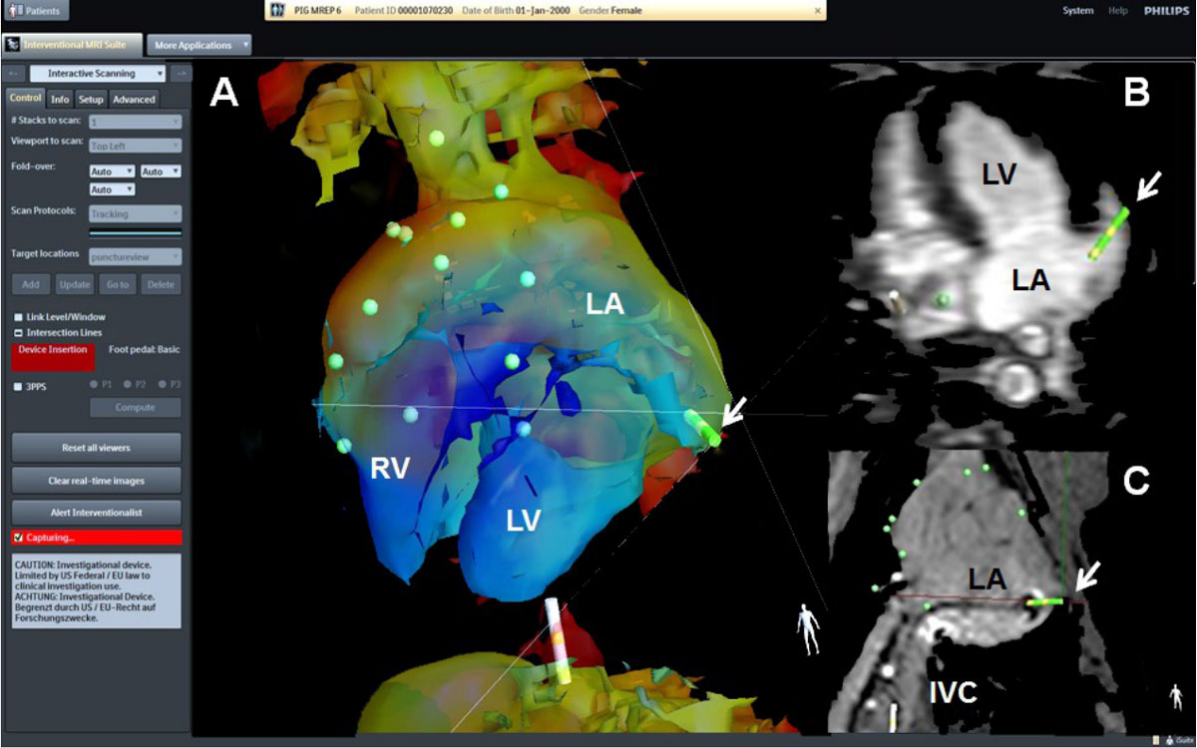
**A: 3D-shell of the advanced navigation platform iSuite (Philips) in anterior-posterior orientation used for active tracking (green catheter tip - white arrow) of the IMRICOR (Vision) ablation catheter**. The tip is located in the left atrial appendage (B - white arrow) after successful transseptal puncture. C: Active tracking overlay (green tip - white arrow) on the passively visualized catheter in the IVC and LA.

